# An Integrated Bioinformatics Approach Identifies Elevated Cyclin E2 Expression and E2F Activity as Distinct Features of Tamoxifen Resistant Breast Tumors

**DOI:** 10.1371/journal.pone.0022274

**Published:** 2011-07-15

**Authors:** Lei Huang, Shuangping Zhao, Jonna M. Frasor, Yang Dai

**Affiliations:** 1 Department of Bioengineering, University of Illinois at Chicago, Chicago, Illinois, United States of America; 2 Department of Physiology and Biophysics, University of Illinois at Chicago, Chicago, Illinois, United States of America; Kyushu Institute of Technology, Japan

## Abstract

Approximately half of estrogen receptor (ER) positive breast tumors will fail to respond to endocrine therapy. Here we used an integrative bioinformatics approach to analyze three gene expression profiling data sets from breast tumors in an attempt to uncover underlying mechanisms contributing to the development of resistance and potential therapeutic strategies to counteract these mechanisms. Genes that are differentially expressed in tamoxifen resistant vs. sensitive breast tumors were identified from three different publically available microarray datasets. These differentially expressed (DE) genes were analyzed using gene function and gene set enrichment and examined in intrinsic subtypes of breast tumors. The Connectivity Map analysis was utilized to link gene expression profiles of tamoxifen resistant tumors to small molecules and validation studies were carried out in a tamoxifen resistant cell line. Despite little overlap in genes that are differentially expressed in tamoxifen resistant vs. sensitive tumors, a high degree of functional similarity was observed among the three datasets. Tamoxifen resistant tumors displayed enriched expression of genes related to cell cycle and proliferation, as well as elevated activity of E2F transcription factors, and were highly correlated with a Luminal intrinsic subtype. A number of small molecules, including phenothiazines, were found that induced a gene signature in breast cancer cell lines opposite to that found in tamoxifen resistant vs. sensitive tumors and the ability of phenothiazines to down-regulate cyclin E2 and inhibit proliferation of tamoxifen resistant breast cancer cells was validated. Our findings demonstrate that an integrated bioinformatics approach to analyze gene expression profiles from multiple breast tumor datasets can identify important biological pathways and potentially novel therapeutic options for tamoxifen-resistant breast cancers.

## Introduction

Estrogen action through estrogen receptor alpha (ER) is a critical regulator of breast cancer cell proliferation and survival. Tamoxifen is an ER antagonist that competitively inhibits the interaction of estrogen with ER and represses ER activity [1], [2], [3]. Tamoxifen has been the primary therapeutic choice in both early and advanced ER positive breast cancer patients since the 1970s. Unfortunately, up to 50% of patients with metastatic disease do not respond to first-line treatment with tamoxifen, and many who receive it as adjuvant therapy experience relapse despite an initial response. Understanding mechanisms by which resistance develops is an important task, which could lead to new therapeutic strategies to combat tumors resistant to endocrine therapy.

Recently, microarray gene expression profiling of ER+ breast tumors has been used to identify gene signatures for prediction of clinical outcome of patients treated with tamoxifen [4], [5], [6], [7], [8]. For example, a 36-gene signature has been derived that can correctly classify up to 80% of patients into relapse or relapse-free groups [7]. Similarly, a 44-gene signature and a 181-gene signature of tamoxifen responsiveness have also been developed from profiling different tumor sets [5], [8]. These gene expression studies were primarily focused on the identification of gene signatures associated with disease progression and clinical outcomes. Therefore, genes in the signatures are not necessarily directly involved in mediating sensitivity to tamoxifen or regulating tumor growth. Furthermore, the analyses of molecular functions of these signature genes have provided only limited insight into underlying mechanisms related to the treatment failure. For example, a preliminary functional analysis of the 36-gene signature in Chanrion et al. [7] indicates that there were 23 under-expressed and 13 were over-expressed genes in tumors from patients with relapse compared to tumors that were relapse free. The under-expressed genes were involved in cellular adhesion or invasion, immune responses, and ER negative regulation, whereas the over-expressed genes were involved in control of mitosis and cell cycle, DNA replication, DNA repair. The 44-gene signature was derived from a set of 81 DE genes that are involved in estrogen action, apoptosis, and extracellular matrix based on functional annotation [8]. On the other hand, the 181-genes in the signature developed by Loi et al. [5] was created from 13 biological clusters determined in the context of a curated list of published molecular interactions by Ingenuity Pathways Analysis (IPA). These clusters represent biological functions such as cell cycle, cell death, DNA repair and cancer inflammation among others. However, whether these functions were represented by the over- or under-expressed genes in the tamoxifen resistant tumors was not clear. Also of note, the three published gene signatures are comprised of distinctly different sets of genes with a small overlap, which presents challenges in deriving any potential mechanisms that may underlie the development of tamoxifen resistance.

We therefore undertook a systematic analysis of three publically available microarray data sets to better understand the biological mechanisms that may contribute to a tamoxifen resistant phenotype [5], [6], [7]. Interestingly, there was little overlap between the three datasets in terms of individual genes that are differentially expressed in tamoxifen resistant vs. sensitive tumors. However, a variety of bioinformatics analyses revealed several functional commonalities in these gene sets, including enhanced cell cycle potential, elevated activity of the target genes of the E2F family of transcription factors, and a number of small molecules that can reverse expression of genes associated with tamoxifen resistance. Finally, we validated the functionality of three small molecules from the phenothiazine family of anti-psychotic drugs to down-regulate cyclin E2 expression and inhibit proliferation of tamoxifen resistant breast cancer cells. Taken together, our findings demonstrate that an integrated approach to analyzing disparate datasets can produce valuable biological information and lead to potential novel therapeutic strategies for drug resistant breast cancers.

## Materials and Methods

### Breast tumor microarray data sets

Datasets used in this work were selected from three breast cancer microarray studies published previously [5], [6], [7] and the raw data were downloaded from Gene Expression Omnibus [9] (accession numbers GSE6532, GSE9195 and GSE9893). Briefly, datasets GSE6532 and GSE9195 consist of gene expression profiles of early stage breast cancer tumors diagnosed between 1980 and 1995 in the John Radcliffe Hospital, Oxford, United Kingdom and Guys Hospital, London, United Kingdom and Uppsala University Hospital, Uppsala, Sweden, respectively. All tumors were required to be ER positive and had received tamoxifen only as adjuvant treatment. Dataset GSE9893 contains gene expression profiles on breast tumors obtained from patients between 1989 and 2001 at the Cancer Research Center of Val d'Aurelle in Montpellier, the Bergonié Institute of Bordeaux, or the Department of Obstetrics and Gynecology of Turin. The patients received tamoxifen treatment for 5 years after surgery. Some of them also received adjuvant radiotherapy. Tumors from each study were classified as tamoxifen sensitive if patients were relapse-free for 5 years or greater or tamoxifen resistant if relapse occurred within 5 years [10]. The 5-year cut-off is based on the criterion published in [10], [11].

### Gene expression analysis

Gene expression analysis was performed using packages in Bioconductor [12]. For each microarray dataset, the probe set intensities were normalized and summarized using the Robust Multichip Averaging algorithm [13] with quantile normalization in rma package. Affymetrix detection calls were obtained to remove low quality probe sets. A second procedure was applied to filter out the least variable probe sets using the percentile of the distribution of coefficient of variability values. The threshold for this filtering was set based on the platform using 0.5 for GSE6532 and GSE9195, and 0.8 for GSE9893. The custom CDF file [14] was used for probe set definition for datasets based on Affymetrix platforms. For the dataset based on 70-mer oligonucleotide microarray, the 22,680 oligonucleotide probes (Oligo Set™ for the Human Genome Version 2.1.3, Qiagen-Operon) representing 21,329 human specific genes were used. The DE genes between the tamoxifen resistant and sensitive tumors were identified using limma package with the Benjamini–Hochberg procedure for multiple test adjustment [15]. The adjusted P-value threshold was set at 0.05.

### Gene function enrichment analysis

For each microarray dataset, over-expressed and under-expressed genes in tamoxifen resistant compared to sensitive tumors were tested for enrichment of functional annotation categories using tools in the Database for Annotation, Visualization and Integrated Discovery (DAVID) (v6.7) [16]. The P-values for the functional annotation enrichment were corrected by the Benjamini–Hochberg method for multiple testing and the significance threshold for the adjusted P-values was set at 0.1.

### Gene set enrichment analysis (GSEA)

GSEA can detect pathways or gene sets whose expression levels are different between two phenotypes based on entire microarray profiles (not just DE genes) using pre-defined gene sets [17]. Two categories of pre-defined gene sets were selected for analysis. One was the canonical pathway gene sets collected from online pathway databases, biomedical literature, and published mammalian microarray studies. The other was a collection of transcription factor target gene sets, which contain genes that share a transcription factor binding site defined in the TRANSFAC (ver7.4) database [18]. These gene sets are available from the Molecular Signature Database (MSigDB) [19]. The gene sets included in our analysis were limited to those with size between 10 and 500 genes. Permutation was carried out 10,000 times using default weighted enrichment statistic and a signal-to-noise metric to rank genes according to their differential expression level across tamoxifen resistant and sensitive tumors. Gene sets with nominal P-value<0.05 were selected.

### Enrichment analysis of differentially expressed genes in breast cancer subtypes

A compendium of 1211 breast cancer microarray expression profiles was used for the enrichment analysis of the DE genes identified by our study in the breast cancer subtypes [20]. These tumors were classified into distinct subtypes based on available tumor annotations. There are 441 tumors in Luminal A, 121 tumors in Luminal B, 136 tumors in normal-like, 152 in Her2, and 279 in basal-like subtypes. The expression data, which were downloaded from the author's website, have been log2-transformed [20]. Gene expression levels are represented relative to the mean of each gene, which was calculated from all samples in the compendium. The enrichment analysis of DE gene sets between tamoxifen resistant and sensitive tumors in the breast cancer subtypes was conducted in two steps. First, for each tumor in the compendium, the up-regulated genes with an expression level greater than 2 and the down-regulated genes with an expression level less than −2, relative to the mean expression of each gene across the compendium, were identified. The enrichment of over-expressed genes from the tamoxifen resistant tumors in the up-regulated gene set from the compendium was analyzed using the hypergeometric test. Similarly, the enrichment of under-expressed genes from the tamoxifen resistant tumors in the down-regulated gene set from the compendium was also analyzed. The significantly enriched tumors were identified using a threshold of P<0.05. Next, the number of enriched tumors in each breast cancer subtype was assigned a P-value according to the hypergeometric test with a threshold of P<0.01 for significance.

### Survival analysis

The principal component analysis (PCA) has been performed for the untreated Luminal A and Luminal B tumors in the compendium set using each of the three DE gene sets (genes not presented in the compendium were excluded). The hierarchical clustering was based on the weights of expression on the top principal components which account for more than 48% of the variance. The Kaplan-Meier estimates were used to compute the survival curves. The packages in R were used for the above analyses.

### Connectivity Map analysis

Entrez gene identifiers of the DE genes in the three microarray datasets were first mapped to Affymetrix HG-U133A probe sets using Affymetrix Human Genome U133A set annotation data implemented in package hgu133a.db in Bioconductor. All mapped probe sets in each individual microarray dataset were then submitted to the Connectivity Map website [21]. A connectivity score based on the Kolmogorov-Smirnov statistic was calculated to estimate the enrichment of both over- and under-expressed query genes in a Connectivity Map instance as described [22]. As the basic unit of data in Connectivity Map, an instance consists of the expression profile of a cell line treated with a compound at a certain concentration and its control pair, as well as a rank-ordered list of all probe sets on the HG-U133A array based on the differential expression level between the pair. The current version of the Connectivity Map (build 02) contains 6,100 such instances representing 5 cultured human cell lines treated with 1,309 compounds. Instances were ranked in descending order of connectivity scores. Multiple independent instances of the same compound with high (or low) rankings indicate positive (or negative) connectivity between the compound and the phenotype represented by the query gene lists. Permutation tests were performed to estimate the significance of the instance sets ranked by the connectivity scores. Compounds were selected from top ranked instance sets with negative connectivity scores at permutation P-value<0.05 for each individual inquiry gene list.

### Cell culture and reagents

MCF-7 breast cancer cell lines that were sensitive to tamoxifen or had developed spontaneous resistance to tamoxifen but remained highly responsive to estradiol (Figure S1) were cultured as previously described [23]. BT474 cells were cultured as previously described [24]. 4-hydroxytamoxifen, trifluoperazine, thioridazine, and prochlorperazine were all obtained from Sigma and used to treat cells, as described in the figure legend, following 3-day incubation in phenol-red free medium supplemented with charcoal-dextran stripped serum.

### Cell viability and proliferation

Cell viability was determined by methylene blue staining [25]. Briefly, each well was rinsed once with PBS and methylene blue staining solution (Hanks' Balanced Salt Solution+1.25% glutaraldehyde+0.6% methylene blue) was added to each well. Following 1 h incubation at 37°C, methylene blue staining solution was removed, and the plates were gently rinsed 3 times in ddH_2_O. Elution solution (50% ethanol+49% PBS+1% acetic acid) was added to each well and subsequently incubated for 20 min at room temperature with gentle agitation. Absorbance was read on a microplate reader at a wavelength of 562 nm and viability was calculated as a percentage of control cells. To study the specific effects of phenothiazines on proliferation, a BrdU assay was carried according to the manufacturer's instructions (Millipore, Billerica, MA). For this assay, MCF 7 cells were treated with phenothiazines for 48 hr with BrdU applied to the cells for the final 24 hours of treatment. Cells were fixed for 30 min and then incubated with anti-BrdU antibody for 1 hour. The goat anti-mouse antibody conjugated to peroxidase was added for 30 min. After washing, cells were incubated with peroxidase substrate for 30 min. Absorbance, correlating to BrdU uptake by the cells, was read at 450 nm wavelength on an automated plate-reader.

### Cyclin E2 mRNA expression

Following treatment of tamoxifen-resistant MCF-7 cells with phenothiazines for 24 hr, total RNA was isolated and Cyclin E2 mRNA was examined by QPCR as previously described [23] using primers specific for Cyclin E2: forward: 5′-GACGGAATCCCCCCAAGA-3′, reverse: 5′-TTTTTTGACATCCTGGGTAGTTTTC-3′. The expression of cyclin E2 following treatment relative to vehicle treated control cells was determined by the ΔΔCT method from three independent experiments.

## Results

### Differentially expressed genes in tamoxifen resistant vs. sensitive breast tumors from three microarray datasets

**Figure 1:**
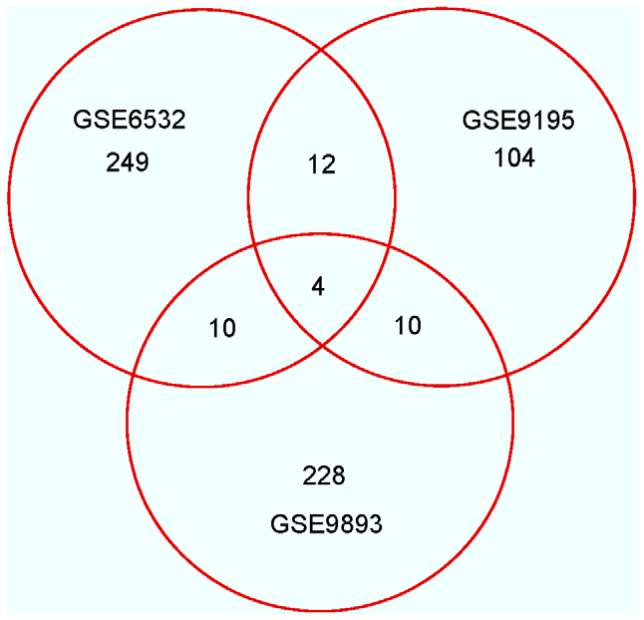
Venn diagram showing overlap between three sets of differentially expressed genes. The numbers of differentially expressed genes, which were either over-expressed or under-expressed in tamoxifen-resistant compared to sensitive tumors, are 275 for GSE6532, 130 for GSE9195, and 252 for GSE9893.

Three sets of microarray gene expression profiles from ER positive breast tumors were obtained from Gene Expression Omnibus as described in Materials and Methods. The numbers of tumors included from each dataset along with the information on the microarray platforms are shown in Table 1 based on the 5-year cut-off for tamoxifen resistance. After filtering out non-variable genes from each dataset, the total numbers of genes analyzed were 3,064, 4,870 and 2,366 for GSE6532, GSE9195 and GSE9893, respectively. The numbers of DE genes, which were either over-expressed or under-expressed in tamoxifen resistant compared to sensitive tumors, are 275 for GSE6532, 130 for GSE9195, and 252 for GSE9893. (A 10-year cut-off was also considered, but the dataset GSE6532 did not provide any differentially expressed genes at FDR = 0.05 following the same analysis protocol). The full list of the DE genes can be found in Table S1. The Venn diagram of overlap of the DE genes among these three datasets is shown in Figure 1. Surprisingly, there are only four genes common in all three sets of DE genes. These are chemokine C-X3-C motif receptor 1 (CX3CR1), which is under-expressed in tamoxifen resistant tumors; cyclin E2 (CCNE2), kinesin family member 4A (KIF4A) and non-SMC condensin I complex, and subunit G (NCAPG), which are over-expressed in tamoxifen resistant tumors. Furthermore, there was very little overlap between any two data sets with just 16 common between GSE6532 and GSE9195, 14 common between GSE9195 and GSE9893, and 14 common between GSE6532 and GSE9893. These findings suggest that either the underlying biology is very different among these three sets of tumors or that some technical aspect of performing the microarrays captured unique sets of DE genes.

**Table 1:** Characterization of the data sets from tamoxifen resistant and sensitive breast tumors used in this study.

		Number of tumors		Number of differentially expressed genes		
Data source	Platform	Tamoxifen resistant	Tamoxifen sensitive	Over-expressed in tamoxifen resistant	Under-expressed in tamoxifen resistant	Total number of genes
GSE6532	Affymetrix HG-U133A	85	91	173	102	275
GSE9195	Affymetrix HG-U133 Plus 2.0	26	138	86	44	130
GSE9893	Qiagen-Operon Oligo Set 2.1.3	49	98	118	134	252

### Common biological pathways identified in tamoxifen resistant tumors

To examine whether the datasets in fact represent biologically different phenotypes, a number of approaches were utilized to compare the three sets of DE genes. First, a functional analysis was performed for each individual dataset using the tools in DAVID. This analysis allows for the identification of particular biological processes or pathways based on functional annotation categories that are over-represented in a set of genes. The three sets of genes that are over-expressed in tamoxifen resistant tumors share a large number of enriched Gene Ontology (GO) terms (Table 2). Specifically, the GO terms in common are mainly associated with cell cycle and DNA replication, suggesting elevated cell proliferative capacity in tamoxifen resistant compared to sensitive tumors. On the other hand, there are no common GO terms shared in the three sets of under-expressed genes in tamoxifen resistant tumors. The entire list of enriched GO terms for each dataset is provided in Table S2. These findings suggest that although there is little overlap of individual genes among the tamoxifen resistant and sensitive tumors, there may be similar underlying biological mechanisms represented in these data sets.

**Table 2:** Common enriched GO terms in the three over-expressed gene sets in tamoxifen resistant tumors.

Ontology	Accession number	Synonyms
Biological process	GO:0000087	M phase of mitotic cell cycle
	GO:0000278	mitotic cell cycle
	GO:0000279	M phase
	GO:0000280	nuclear division
	GO:0006259	DNA metabolic process
	GO:0006260	DNA replication
	GO:0007049	cell cycle
	GO:0007067	mitosis
	GO:0007346	regulation of mitotic cell cycle
	GO:0010564	regulation of cell cycle process
	GO:0022402	cell cycle process
	GO:0022403	cell cycle phase
	GO:0048285	organelle fission
	GO:0051301	cell division
	GO:0051726	regulation of cell cycle
Cellular component	GO:0000777	condensed chromosome kinetochore
	GO:0000779	condensed chromosome, centromeric region
	GO:0000793	condensed chromosome
	GO:0005694	chromosome
	GO:0005819	spindle
	GO:0015630	microtubule cytoskeleton
	GO:0043228	non-membrane-bounded organelle
	GO:0043232	intracellular non-membrane-bounded organelle
	GO:0044427	chromosomal part
	GO:0044430	cytoskeletal part
Molecular function	GO:0001882	nucleoside binding
	GO:0001883	purine nucleoside binding
	GO:0005524	ATP binding
	GO:0030554	adenyl nucleotide binding
	GO:0032559	adenyl ribonucleotide binding

To infer the possible mechanisms underlying the altered gene expression profiles in tamoxifen resistant tumors, we performed GSEA [17] to determine if specific pre-defined gene sets were significantly enriched in tamoxifen resistant tumors. Four canonical pathways, DNA replication reactome, G1_to_S cell cycle reactome, HSA04110 cell cycle, and purine metabolism, were found to be commonly enriched pathways among all three datasets. Over-expressed genes, POLE3, PRIM1, RFC3, RFC4, RFC5 (in GSE6532), DBF4, GMNN, FEN1, MCM10, PSMD12 (in GSE9195) and CCNA2, CDC6, CDT1, MCM2, MCM4 (in GSE9893), are involved in DNA replication. Other over-expressed genes such as CCNA2 (in GSE9893), CCNE2 (in GSE6532, GSE9195 and GSE9893), and CCNB2, CDC2, KIF2C, RRM2 (in GSE9195 and GSE9893) are associated with the cell cycle pathways. This finding suggests that tamoxifen resistant tumors likely have an intrinsically elevated level of cell proliferation. This concept was further supported by GSEA with transcription factor (TF) target gene sets. The analysis is designed to examine whether activity of a specific TF is significantly associated with given gene expression data. In the case of tamoxifen resistant tumors, target genes for the TFs TFDP1, TFDP2, E2F1, and E2F4 were found to be significantly enriched in all three microarray datasets (Table 3). TFDP1 and TFDP2 are transcriptional coactivators that can stimulate E2F-dependent transcription of a number of genes whose products are involved in control of cell-cycle progression from G1 to S phase, DNA replication, and p53-dependent/independent apoptosis [26]. In our analysis the expression levels of all four TFs are not significantly altered between tamoxifen resistant and sensitive tumors. However, genes known to be regulated by these TFs were over-expressed in tamoxifen resistant tumors; for example, FBXO5 (in GSE6532), TOPBP1 (in GSE9195), MCM2, MCM4, CDC6 (in GSE9893), and CDC2 (in GSE9195 and GSE9893). All enriched pathways and TF target gene sets are given in Table S3. Thus, our findings from the enrichment analyses of functional annotation, canonical pathway, and TF target gene sets are all consistent with the concept that elevated cell proliferation pathways are a hallmark of tamoxifen resistant breast tumors.

**Table 3:** Transcription factor (TF) binding sites and the putative TFs that are common among all three microarray datasets of tamoxifen resistant tumors.

Name of response element	Putative TF associated with enriched target genes
V$E2F_Q3_01	TFDP1: transcription factor Dp-1
V$E2F_Q4_01	TFDP1: transcription factor Dp-1
V$E2F_Q6	E2F1: E2F transcription factor 1
V$E2F_Q6_01	TFDP1: transcription factor Dp-1
V$E2F1_Q3	E2F1: E2F transcription factor 1
V$E2F1_Q6	E2F1: E2F transcription factor 1
V$E2F1_Q6_01	E2F1: E2F transcription factor 1
V$E2F1DP1_01	E2F1: E2F transcription factor 1; TFDP1: transcription factor Dp-1
V$E2F1DP2_01	E2F1: E2F transcription factor 1; TFDP2: transcription factor Dp-2 (E2F dimerization partner 2)
V$E2F4DP1_01	E2F4: E2F transcription factor 4, p107/p130-binding; TFDP1: transcription factor Dp-1
V$E2F4DP2_01	E2F4: E2F transcription factor 4, p107/p130-binding; TFDP2: transcription factor Dp-2 (E2F dimerization partner 2)

### Enrichment of the differentially expressed genes in breast cancer subtypes

**Figure 2:**
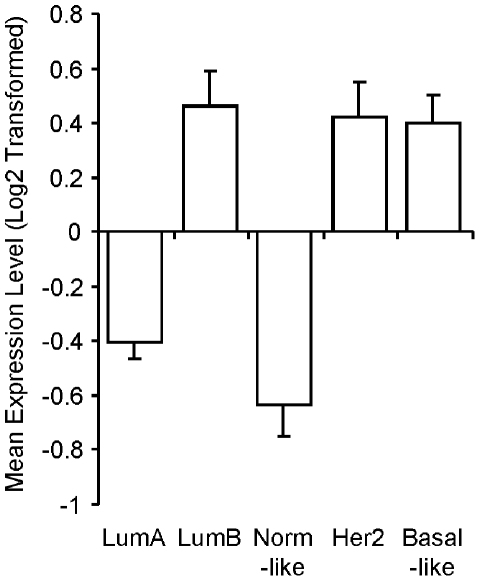
Expression level of Cyclin E2 mRNA in breast tumors from different intrinsic subtypes. The mean expression level of Cyclin E2 was determined in breast tumors of different intrinsic subtypes as described in Materials and Methods. The Log2 transformed mean expression level is relative to the mean expression level in all tumors in the breast tumor compendium.

To explore whether the DE genes in tamoxifen resistant vs. sensitive tumors were enriched in specific intrinsic subtypes of breast cancer, a breast cancer compendium was utilized as described in Materials and Methods. Table 4 shows that genes over-expressed in tamoxifen resistant tumors are enriched in the Luminal B tumor subtype for two of three datasets, in the Basal-like tumor subtypes for all three datasets, and the Her2 subtype for only 1 dataset. In contrast, genes under-expressed in tamoxifen resistant tumors are enriched in Luminal A subtype tumors for all three datasets. In addition, the average expression level of CCNE2 is consistently higher in Luminal B, Basal-like, and Her2 tumor subtypes than average, and lower than average in Luminal A and Normal-like tumor subtypes (Figure 2). In addition, the transcription factor target gene sets of E2F1 and TFDP1 used in GSEA were also enriched in Luminal B tumor subtype (P = 0.014 and P = 0.006, respectively) while TFDP2 and E2F4 target gene sets showed no enrichment in Luminal B subtype (P = 0.302 and P = 0.302, respectively) (Table S4). These results indicate that tumors from the Luminal B intrinsic subtype share a similar highly proliferative phenotype with tamoxifen resistant tumors.

**Table 4:** P-values of gene set enrichment analysis on the breast cancer compendium.

Subtypes and numbers in BC compendium	Over-expressed genes in tamoxifen resistant tumors			Under-expressed genes in tamoxifen resistant tumors		
	GSE6532	GSE9195	GSE9893	GSE6532	GSE9195	GSE9893
Luminal A (411)	1.000	1.000	1.000	**<0.001**	**0** *	**0** *
Luminal B (212)	**<0.001**	0.217	**<0.001**	1.000	0.770	0.523
Normal-like (136)	1.000	1.000	1.000	**<0.001**	0.913	0.414
Her2 (152)	0.292	0.276	**0** *	1.000	1.000	1.000
Basal (270)	**0.002**	**0** *	**<0.001**	1.000	1.000	1.000

*P-values are smaller than 1.00E-15.

**Figure 3:**
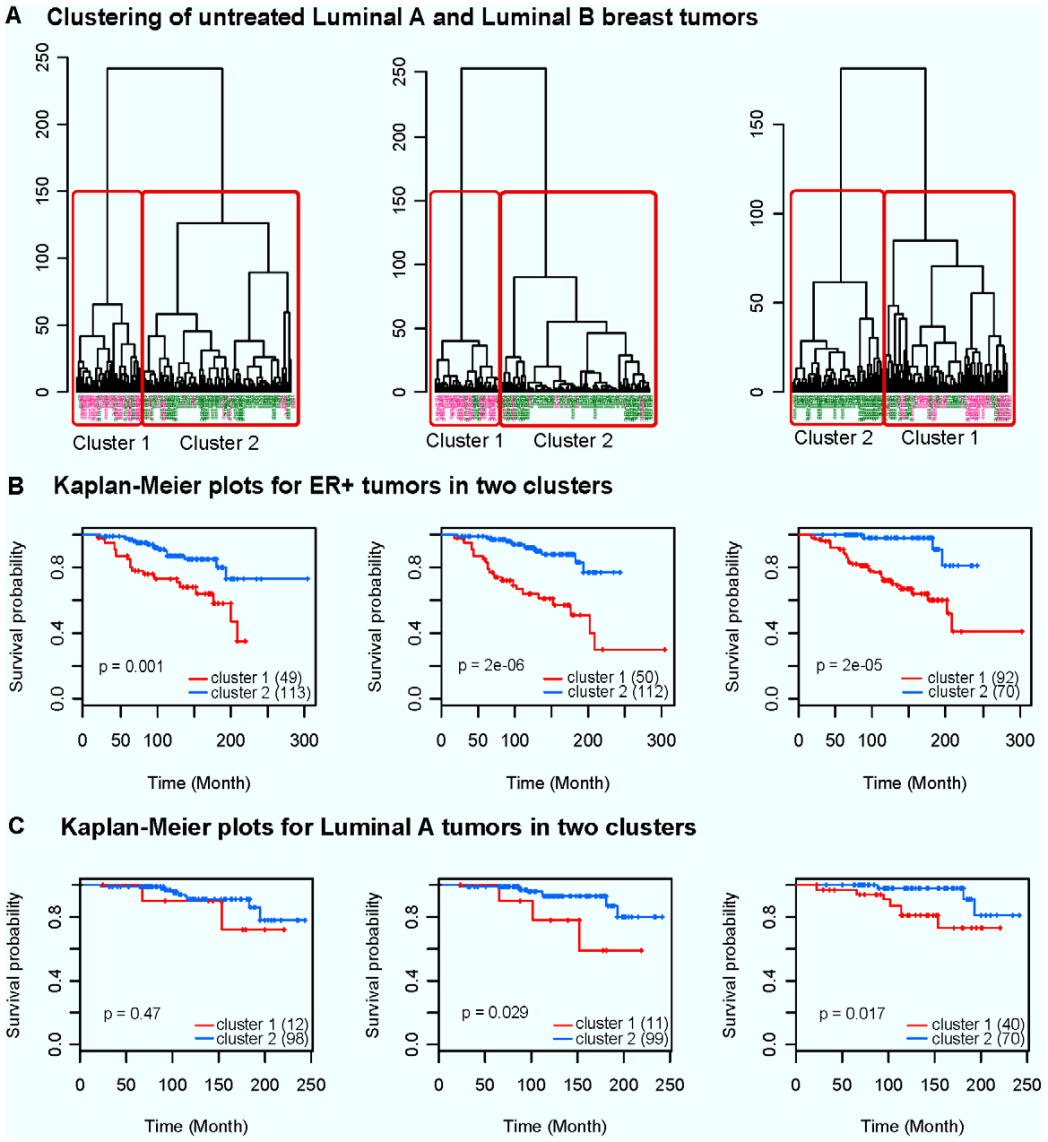
Results of clustering and survival analysis. (A) Clustering results of untreated Luminal A and Luminal B breast tumors from the compendium gene expression profile using principle components of the DE genes identified in GSE6532 (left), GSE9195 (middle) and GSE9893 (right). Green: luminal A tumors; Pink: luminal B tumors; (B) Kaplan-Meier estimation of survival for stratified ER+ tumors using the DE genes identified in GSE6532 (left), GSE9195 (middle) and GSE9893 (right); the number in the parentheses is the number of ER+ tumors in each cluster. (C) Kaplan-Meier estimation of survival for stratified Luminal A tumors using the DE genes identified in GSE6532 (left), GSE9195 (middle) and GSE9893 (right); the number in the parentheses is the number of Luminal A tumors in each cluster.

We further investigated whether using the DE genes between tamoxifen resistant and sensitive tumors can stratify ER+ tumors with distinct outcome. For this purpose, we selected a subset of untreated ER+ tumors (110 Luminal A and 52 Luminal B) with survival data from the compendium. We retrieved the expression values of the DE genes for these tumors. A PCA analysis was performed on their gene expression values corresponding to each DE set as described in Materials and Methods. Hierarchical clustering was performed using each of the three DE gene set (Figure 3A). We stratified the tumors into two clusters. Most of the Luminal B tumors appeared in cluster 1, but mixed with some Luminal A tumors, largely seen in the clustering corresponding to the third gene set. The Kaplan-Meier analysis shows that the two clusters are significantly different in outcomes, with P = 0.001, P = 2e-06 and P = 2e-05, respectively for the three DE gene sets (Figure 3B). This indicates the Luminal B subtypes has a similarly intrinsic molecular profile to that of the tamoxifen resistant tumors. Although some Luminal B tumors were present in cluster1 for all three datasets, the two Luminal B subgroups did not show significantly different outcome (data not shown). However, it was surprising to observe that in two cases the luminal A tumors stratified into the two clusters demonstrated a significant difference in outcome (P = 0.47, P = 0.029 and P = 0.017, respectively) (Figure 3C). It appears that within the Luminal A tumors, there is a subgroup of tumors possessing a molecular profile similar to that of the tamoxifen resistant tumors. These results may appear to be contradictory to the enrichment findings in Table 4, as the Luminal A subtype was not significantly enriched in the over-expressed genes in the tamoxifen resistant tumors. However, this does not exclude the existence of some Luminal A tumors being actually enriched by the over-expressed genes.

### Small molecules that reverse proliferation of tamoxifen resistant cell lines

In an attempt to link the gene expression profiles derived from the tamoxifen resistant and sensitive tumors to small molecules that may alter the profiles, and potentially tamoxifen resistance, an analysis was performed using the Connectivity Map [22], [27] as described in Materials and Methods. The Connectivity Map allows researchers to screen compounds by comparing a ranked list of genes based on the association to a disease phenotype with the expression profiles derived from the several types of cell lines treated with compounds. If a small molecule produces the gene expression pattern opposite to those observed between tamoxifen resistant and sensitive breast cancers, then a negative score will be assigned based on the Connectivity Map analysis. The molecules with negative scores will be considered to have potential to reverse the tumor expression pattern if the tumors were treated with the molecules. The top-ranked compound/cell combinations with negative connectivity scores that are common among the three tamoxifen resistant gene sets are listed in Table 5. The entire list of enriched compounds for each individual microarray dataset can be found in Table S5.

**Table 5:** Common top ranked compounds with expression profiles opposite to those of the tamoxifen resistance tumors.

		GSE6532			GSE9195			GSE9893		
Compound	Cell Line	Enrich Score	Rank	P *	Enrich Score	Rank	P *	Enrich Score	Rank	P *
trichostatin A	PC3	−0.372	1	0	−0.844	1	0	−0.627	2	0
trichostatin A	MCF7	−0.281	12	0	−0.59	3	0	−0.58	3	0
LY-294002	MCF7	−0.374	3	<0.001	−0.412	4	0	−0.441	5	0
resveratrol	MCF7	−0.696	9	0.002	−0.767	13	<0.001	−0.551	143	0.031
trifluoperazine	MCF7	−0.53	24	0.007	−0.641	14	0	−0.642	11	0
thioridazine	PC3	−0.671	29	0.009	−0.896	6	<0.001	−0.872	6	<0.001
DL-thiorphan	MCF7	−0.933	32	0.009	−0.886	73	0.026	−0.905	92	0.018
harmine	MCF7	−0.929	26	0.011	−0.948	31	0.006	−0.927	67	0.011
0297417-0002B	MCF7	−0.928	38	0.011	−0.977	20	0.001	−0.973	21	0.001
chrysin	MCF7	−0.907	51	0.017	−0.943	35	0.007	−0.887	116	0.025
trimethylcolchicinic acid	MCF7	−0.896	62	0.021	−0.894	68	0.022	−0.911	89	0.016
galantamine	MCF7	−0.868	92	0.035	−0.92	46	0.013	−0.916	77	0.014

*P-values of 0 are smaller than 1.00E-15.

**Figure 4:**
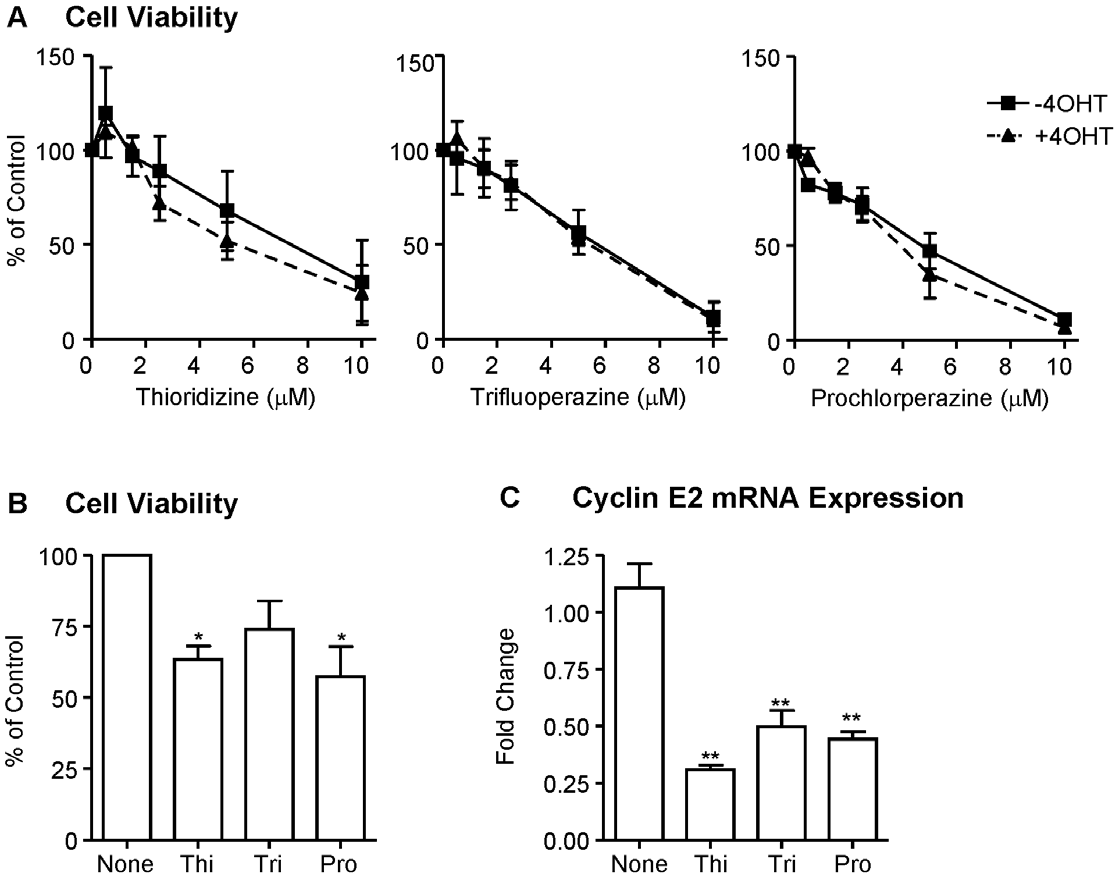
Phenothiazines inhibit proliferation and down-regulate Cyclin E2 expression in tamoxifen-resistant MCF-7 breast cancer cells. (A) Cells were treated for five days with increasing doses of the three phenothiazine compounds in the absence or presence of 1 µM 4-hydroxytamoxifen (4OHT) as indicated. Cell viability was determined by methylene blue staining and expressed as % of vehicle treated control cells. (B) A BrdU assay was carried out after 48 hr of treatment with 5 µM of each phenothiazine drug. (C) Cyclin E2 mRNA levels were determined by QPCR following 24 hr treatment with 5 µM of each phenothiazine as indicated. All data represent the mean +/− SEM from three independent determinations. *, P<0.05, **, P<0.01.

**Figure 5:**
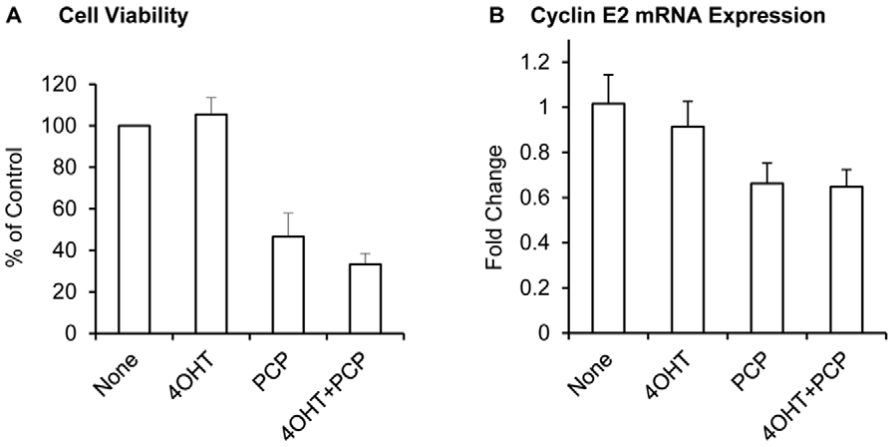
Prochlorperazine inhibits growth of BT474 cells. Cells were treated 5 µM of prochlorperazine for 5 days and cell proliferation was measured by methylene blue staining (A) or for 2 days and cyclin E2 mRNA levels were by QPCR (B).

Among the top-ranked compounds listed in Table 5, three drugs (trifluoperazine, thioridazine, and prochlorperazine) belong to the same structural family of phenothiazine compounds. From the Connectivity Map database, expression of cyclin E2 was found to be lower in MCF-7 and PC3 cells treated with these three compounds than in controls (data not shown). To validate whether these drugs may have any effect on proliferation or gene expression in tamoxifen resistant breast cancer cells, MCF-7 cells that developed spontaneous resistance to tamoxifen were utilized (Figure S1). Cells were treated with increasing concentrations of the three phenothiazines, trifluoperazine, thioridazine, and prochlorperazine in the presence of 4-hydroxytamoxifen (4OHT). After 5 days of treatment, a dose-dependent decrease in the number of viable cells was observed for each of the phenothiazine compounds independent of the presence of 4OHT (Figure 4A). A similar growth inhibitory effect was observed in MCF-7 cells sensitive to tamoxifen (data not shown). A BrdU assay was carried out in the resistant cells after 48 hr of treatment and demonstrates that the growth inhibitory effects of phenothiazines are associated with a reduction in cell proliferation (Figure 4B). To examine the effect of these compounds on cyclin E2 expression, QPCR was carried out. All three phenothiazines down-regulated expression of cyclin E2 mRNA levels within 24 hr of treatment (Figure 4C). To confirm our findings in another cell line, we utilized BT474 cells that over-express HER2 and have previously been shown to be tamoxifen resistant [28], [29]. Treatment with 5 µM of prochlorperazine in the presence or absence of 4OHT significantly inhibited cell proliferation and reduced cyclin E2 mRNA levels (Figure 5). These findings validate the bioinformatics analyses described above and identify a novel class of therapeutic drugs that have the potential to inhibit proliferation of both tamoxifen-sensitive and tamoxifen-resistant breast tumors.

## Discussion

In an attempt to understand more clearly the molecular mechanisms involved in tamoxifen resistant breast tumors, we undertook an integrative bioinformatics analysis approach using three published gene expression datasets from ER positive breast tumors. It should be noted that these datasets have been previously analyzed individually to derive gene signatures for the prediction of tamoxifen responsiveness and clinical outcomes; limited information on functionality of these genes have been provided through gene function annotation or pathway analysis [5], [6], [7]. However, these datasets have not been compared or analyzed together in any comprehensive manner. Our initial analysis revealed only four genes that were differentially expressed between tamoxifen resistant and sensitive tumors and common among all three datasets. Despite this apparent difference in gene expression profiles, a variety of functional analyses revealed a high degree of biological commonality between these tumor sets. This success of our approach may be attributed to the power of GSEA [17], which does not rely on a set of DE genes but rather uses a knowledge-based approach to identify pathways enriched by genes with a moderate but not significant level of differential expression. In addition, gene set enrichment analysis using the pre-defined transcription factor target gene sets enabled the detection of transcription factors whose activity but not expression levels are different in resistant and sensitive tumors. Furthermore, the Connectivity Map analysis allows for the detection of small molecules that are linked to tamoxifen resistance through analysis of similarity or dissimilarity between entire gene expression profiles. Using existing knowledge of pathways and regulation of gene expression, these approaches appear to be more effective compared with any single-gene analysis, which may miss important biologically active pathways.

It should be noted that a different approach can be taken by pooling tumors profiled with the similar platforms, such as the U133A, U133B and U133 Plus 2.0 Affymetrix GeneChips. In fact, we also performed the analysis by pooling tumors in GSE6532 and GSE9195 sets using common probesets between U133A and U133 Plus 2.0 chips. Although a larger common DE gene set was identified, we did not find significant difference in the sets of enriched GO terms and pathways. However, the effectiveness of the pooled analysis has to rely on a proper procedure of batch-effect removal.

A wide variety of proposed mechanisms for tamoxifen resistance have been described [30] but the majority of these have been identified using cell lines that have acquired resistance to tamoxifen over long-term exposure. The gene expression profiles utilized for our study, on the other hand, are taken from primary breast tumors prior to exposure to tamoxifen. Thus, our findings potentially represent de novo or intrinsic molecular mechanisms of resistance. One gene over-expressed in all three datasets, cyclin E2, which is an essential regulator of G1 to S phase transition during the cell cycle, is of particular interest. Previous studies have shown that over-expression of cyclin E2 in cell lines is associated with the development of tamoxifen resistance [31]. In human breast tissue it was found that cyclin E2 levels are elevated in tumors vs. normal tissue [32] and that both cyclin E1 and E2 protein levels are associated with a poor response to tamoxifen [33].

In addition to cyclin E2, the integrated analyses that we performed also identified several pathways and GO terms, in particular related to cell proliferation, that are enriched in all three tamoxifen resistant tumor sets. Furthermore, we find activity of the E2F family of transcription factors is strongly associated with the tamoxifen resistant phenotype in all three datasets. Interestingly, one of the gene expression profiling datasets used in our analysis was previously used to identify elevated c-Myc activity as associated with enhanced proliferation signature and reduced responsiveness to tamoxifen [34]. We have confirmed their findings (Table S3) but were unable to detect an elevated c-Myc signature in the other two datasets examined, which indicates the importance of an integrated analysis for the identification of common mechanisms implied in different microarray datasets. We have detected a strong association between tamoxifen resistance and cell proliferation/cell cycle gene expression signatures, which suggests that tamoxifen resistant tumors display a highly proliferative phenotype compared with tamoxifen sensitive tumors. The examination of genes differentially expressed in tamoxifen resistant vs. sensitive tumors in breast tumor intrinsic subtypes revealed that tamoxifen resistance is highly correlated with the Luminal B and Basal-like subtypes. While Luminal B tumors have a worse outcome than Luminal A tumors in general [35], most likely due to enhanced growth factor signaling [36], a clear association between gene signatures of Luminal subtype and tamoxifen responsiveness has not been made. Our findings support this but the similarity between tamoxifen resistant tumors and both Luminal B and Basal-like subtypes also suggests the possibility that the increased proliferation signatures in each of these tumor types could be an underlying factor. In addition, our analysis also found that the Luminal A tumors can be stratified into two subgroups using the DE genes and that these two subgroups have distinctly different outcomes, suggesting that Luminal A and Luminal B subtypes represent a heterogeneous populations and understanding the relationship between Luminal subtype and tamoxifen responsiveness requires further study.

Using the Connectivity Map analysis we also linked the gene expression profiles of several small molecules to those derived from the three tamoxifen resistant and sensitive tumor datasets. Although the small molecules utilized in the Connectivity Map analysis were presumably tested in MCF-7 cells that are sensitive to tamoxifen, we were able to identify many drugs that induce an opposite gene expression profile of that seen in tamoxifen resistant tumors. The top identified compounds belong to different chemical classes. For example, a HDAC inhibitor (trichostatin A), a PI3K inhibitor (LY294002), natural compounds, such as resveretrol and chrysin, and several drugs, including phenothiazines (trifluoperazine and thioridazine), monoamine oxidase A (MAO-A) inhibitor (harmine), and a colchicine analog (trimethylcolchicinic acid), were all found to produce an opposite gene expression profile in MCF-7 or the prostate cancer cell line, PC3.

Validation studies were carried out on three structurally similar drugs from the phenothiazine family (trifluoperazine, thioridazine, and prochlorperazine) since they were structurally similar and all found to down-regulate cyclin E2, a gene differentially expressed in all three datasets. These drugs were originally designed as anti-malarial drugs but have been shown to act as anti-histamines, anti-emetics, suppressants of psychotic symptoms, and anti-cholinergics. We confirmed that these drugs reduce cyclin E2 gene expression and cell proliferation in MCF-7 cells that are resistant to tamoxifen. The mechanisms by which these drugs act are not fully clear but they are known to inhibit calmodulin and prostaglandin synthesis, both of which have the potential to impact on estrogen receptor (ER) function and alter response to endocrine therapy [37], [38]. In fact, early studies suggested that the anti-proliferative capacity of trifluoperazine correlated with its ability to antagonize calmodulin activity and that calmodulin inhibitors in combination with tamoxifen may have synergistic activity [39]. Other studies have also suggested an interaction between phenothiazines and tamoxifen in inducing apoptosis in cancer cells [40], [41]. However, our studies did not show any interaction between tamoxifen and phenothiazines in any of the cell types or assays tested. Alternatively, these drugs may act independently of estrogen receptor and have a general anti-proliferative effect on breast cancer cells, as suggested by the fact that they also inhibit proliferation of tamoxifen sensitive MCF-7 cells. Previous studies have suggested that these drugs may sensitize breast tumors with a multi-drug resistance phenotype [42], induce apoptosis, potentially in combination with tamoxifen [40], and/or promote autophagy [43], [44].

Exploration of phenothiazines as agents to inhibit growth of tamoxifen-resistant and sensitive, breast cancer cells requires further study. Also, since these agents have been used in vivo testing their ability to reduce tumor burden in preclinical xenograft models also warrants further investigation.

In conclusion, our findings demonstrate that an integrated bioinformatics approach to analyze gene expression profiles from multiple breast tumor datasets can identify important biological pathways and potential novel therapeutic options for tamoxifen-resistant breast cancers.

## Supporting Information

Figure S1 **Growth Response of MCF-7 Cells to Estradiol and Tamoxifen.** MCF-7 cells that spontaneously developed resistance to tamoxifen were cultured for 5 days in the presence of 10 nM E2, 1 mM 4-hydroxytamoxifen, or both. Cell proliferation was measured by methylene blue assay and calculated as percentage of control. Data shown are mean +/− SE for 3 independent replicates.
(TIF)

Table S1 Differentially expressed genes between tamoxifen resistant and sensitive tumors in each data set (adjusted p value<0.05).
(XLS)

Table S2 GO term functional annotation chart for differentially expressed genes in each data set using DAVID.
(XLS)

Table S3 List of the canonical pathways and transcription factor target (TFT) gene sets enriched in tamoxifen resistant tumors in each data set through GSEA (p<0.05).
(XLS)

Table S4 Transcription factor target (TFT) gene set enrichment in the Breast Cancer Compendium.
(XLS)

Table S5 List of enriched compounds through the Connectivity Map analysis.
(XLS)
